# Triple-tyrosine kinase inhibition by BIBF1000 attenuates airway and pulmonary arterial remodeling following chronic allergen challenges in mice

**DOI:** 10.1186/s40001-023-01037-2

**Published:** 2023-02-09

**Authors:** Malarvizhi Gurusamy, Saeed Nasseri, Dileep Reddy Rampa, Huiying Feng, Dongwon Lee, Anton Pekcec, Henri Doods, Dongmei Wu

**Affiliations:** 1 Department of Bio-Nanotechnology and Bio-Convergence Engineering, Chonbuk National University, Jeonju, South Korea; 2 Department of Research, Mount Sinai Medical Center, Miami Beach, FL USA; 3 Research Beyond Borders, Boehringer Ingelheim Pharma GmbH & Co. KG, Biberach, Germany; 4 Present Address: Cellular and Molecular Research Center, Birjand University of Medical Sciences, Birjand, Iran

**Keywords:** Tyrosine kinase, Airway inflammation, Asthma, Airway remodeling, Mucus

## Abstract

**Background:**

Airway remodeling is an important pathological feature of chronic airway diseases, which leads to a progressive decline in lung function. The present study examined the anti-remodeling and anti- inflammatory effect of BIBF1000, a triple-tyrosine kinase inhibitor that targets VEGF, PDGF, and FGF receptor signaling in a mouse model of repeated ovalbumin (OVA) challenges.

**Methods:**

Female Balb-c mice were immunized intraperitoneally on days 0 and 12 with 50 µg ovalbumin plus 1 mg of Al(OH)3 in 200 μl saline. Intranasal OVA challenges (20 µg/50 µl in PBS) were administered on days 26, 29, and 31, and were repeated twice a week for 3 months. Animals received vehicle or BIBF1000 (25 mg/kg, b.i.d.) through gavage from day 26 to the end of fourth month. On day 120, bronchoalveolar lavage (BAL) and lung tissue were collected for biochemical and immunohistological analysis.

**Results:**

Compared to vehicle controls, treatment with BIBF1000 reduced the numbers of BAL eosinophils, macrophages, neutrophils, and lymphocytes by 70.0%, 57.9%, 47.5%, and 63.0%, respectively, and reduced IL-5 and IL-13 in BAL. Treatment with BIBF1000 reduced airway mucus secretion, peribronchial fibrosis, small airway, and pulmonary arterial wall thickness, compared to vehicle controls. Furthermore, treatment with BIBF1000 also reduced the expression of inflammatory mediators (TNF-α, IL-1β, IL-5, IL-13, MMP-2, MMP-9, COX-2, and iNOS) and inhibited ERK and AKT phosphorylation.

**Conclusions:**

The protective effect afforded by triple-tyrosine kinase inhibition with BIBF1000 in reducing allergen-induced airway and arterial remodeling was associated with down-regulation of inflammatory mediators, as well as inhibition of ERK and AKT signaling pathways.

## Introduction

Airway remodeling is an important pathological feature of chronic lung inflammatory diseases, including asthma, chronic obstructive pulmonary disease (COPD), and pulmonary arterial hypertension (PAH), which leads to a progressive decline in lung function [1–4]. Airway remodeling refers to structural changes resulting from persistent inflammation in airway tissues, which is characterized by airway smooth muscle hypertrophy and hyperplasia, collagen deposition to sub-epithelial basement membrane, hyperplasia of goblet cells, thickening of airway mucosa, and an increase in vascularity [3, 5]. A variety of growth factors secreted from damaged epithelial and inflammatory cells, including basic fibroblast growth factor (FGF), platelet-derived growth factor (PDGF), vascular endothelial growth factor (VEGF), and transforming growth factor-β (TGF-β) can activate the fibrotic process upon binding to their receptors, resulting in airway wall thickening and increased microvasculature which drive structural changes linked to airway remodeling [6–9]. FGF, PDGF, and VEGF receptors belong to the tyrosine kinase receptor (RTK) family [10]. Activation of RTKs can activate signaling pathways that promote the proliferation of airway smooth muscle cells and fibroblasts, extracellular matrix production and airway inflammation, contributing in many processes of airway remodeling [4, 11–14].

Emerging evidence suggests that RTKs play a critical role in the pathogenesis of chronic airway diseases. For example, VEGF levels in sputum and BALF are increased in asthmatics, and the levels correlate directly with the disease activity [12]. VEGF promotes both airway inflammation and remodeling, and leading to increased vascular permeability in asthma [3, 4, 12]. PDGF receptors (PDGFR) are overexpressed in patients with severe asthma [15], and PDGFR signaling plays a prominent role in airway inflammation and remodeling through altering fibroblast chemotaxis, proliferation, and collagen production [16]. In addition, FGF-2 potentiates the release of TGF-β1 from macrophages, and that FGF-2 and TGF-β1 synergistically stimulate the proliferation of bronchial smooth muscle cell (BSMC) through up-regulation of the PDGFRs, thus contribute to the hyperplastic phenotype of BSMC in remodeled asthmatic airways [13, 17]. Given that multiple RTKs are involved in the pathology of airway remodeling, simultaneous inhibition of multiple RTKS might be an effective approach for anti-remodeling in chronic airway diseases. Nintedanib, a triple RTK inhibitor that blocks PDGFR, VEGFR, and FGFR, exhibits a powerful anti-fibrotic effect, and has been approved by the FDA for treatment of idiopathic pulmonary fibrosis (IPF) [18]. Nintedanib ameliorated airway remodeling in a mouse model of OVA-induced chronic asthma [19]. BIBF1000, an orally active potent inhibitor of the receptor tyrosine kinases for VEGF, FGF, and PDGF, prevents and reverses the progression of severe pulmonary arterial hypertension, inhibits pulmonary arterial neointimal formation, attenuates right heart hypertrophy, and improves survival in an experimental model of PAH [20]. The present study examined the anti-remodeling and anti- inflammatory effect of BIBF1000 in a mouse model of ovalbumin allergen-induced chronic asthma.

## Methods

### Animals

These animal studies were approved by the Institutional Animal Care and Use Committee at Chonbuk National University (CBU 2013-0044). All experiments were conducted in accordance with the National Institutes of Health Guide for the Care and Use of Laboratory Animals.

### Chronic OVA-challenged mouse model

**Fig. 1:**
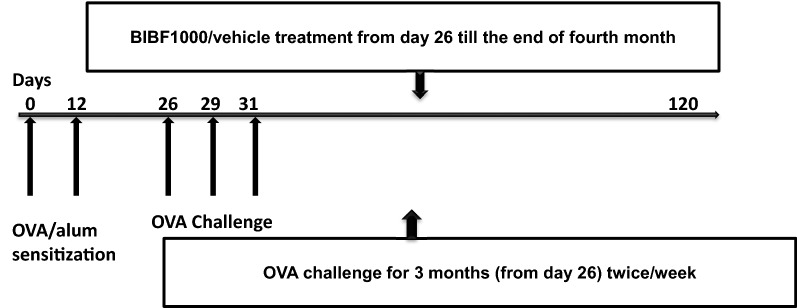
Schematic diagram of experimental design

Female Balb-c mice (8–10 weeks old) were randomly assigned into three study groups: (1) Control, (2) OVA + vehicle, and (3) OVA + BIBF1000. The reason for choosing female mice is based on the previous studies that female mice experience more airway remodeling compared with male mice [21], and that adult women are more susceptible to developing asthma than men and that women display more severe forms of the disease [22]. The study protocol is illustrated in Fig. 1. Briefly, mice were immunized intraperitoneally on days 0 and 12 with 50 µg ovalbumin plus 1 mg of Al(OH)3 in 200ul saline. Intranasal OVA challenges (20 µg/50 µl in PBS) were administered on days 26, 29, and 31, and were repeated twice a week for 3 months. Animals received vehicle (0.1% Natrosol) or BIBF1000 (25 mg/kg, b.i.d., a gift from Boehringer Ingelheim Pharma KG, Biberach, Germany) through gavage from day 26 to the end of fourth month. On day 120, bronchoalveolar lavage (BAL) was collected and analyzed for inflammatory cell influx and biochemical mediators as previously described [23]. Briefly, 0.5 ml of sterile PBS was instilled into the mouse lung via a 20-gauge angiocath and lavaged three times. BAL fluid samples were then pooled and centrifuged, and cell numbers and differentials were assessed. The cell-free BAL fluid was stored at − 80 °C until used.

### Lung histology

Lung tissues were fixed with 10% formalin (Sigma, St. Louis, MO), and embedded in paraffin. Lung tissues were cut in 5 µm-thickness sections and stained with haematoxylin and eosin (H& E, Sigma, St. Louis, MO), periodic acid Schiff (PAS), trichrome stain. For IHC analysis, lung sections were deparaffinized, hydrated, and incubated in 10 mM sodium citrate buffer at 99 °C for 20 min for antigen retrieval. Sections were incubated overnight with a primary antibody (1:350) to one of the following antigens: α-Smooth muscle actin (ab-5694, Abcam, Cambridge, MA), matrix metalloproteinase (MMP)-2 (sc-13594, Aviva Biosystems, San Diego, CA), MMP-9 (sc-21733), inducible nitric oxide synthase (iNOS, sc-7271), proliferating cell nuclear antigen (PCNA, sc-25280), and cyclooxygenase 2 (COX-2, sc-7951) (All from Santa Cruz Biotechnology, Santa Cruz, CA) as previously described [23]. Next, the section was incubated for 1 h with the FITC-labeled goat anti-rabbit IgG secondary antibody (1:300) (sc-2012, Santa Cruz Biotechnology), Alexa Fluor 594, goat, anti-mouse (Catalog# 405326, BioLegend) secondary antibody, or Goat pAb to Rb IgG (Alexa Fluora 594), ab 150084 (abcam) secondary antibody. Sections were counterstained with Ultra Cruz Mounting Medium with 4ʹ, 6-diamidino-2-phenylindole (DAPI; sc-24941, Santa Cruz Biotechnology) and coverslipped. Fluorescent images were taken using the Nikon Eclipse TE2000-U fluorescence microscope (Nikon Corp., Tokyo, Japan) and a Nikon LWD 0.52 digital camera. Two–three lung sections per mouse were analyzed. Images with the same magnification (20x) were analyzed using image J software. Fluorescence intensity was measured based on area, and fluorescence intensity was normalized with control group.

For mucus staining: two to three lung sections from each animal were stained with Periodic Acid of Schiff (PAS) for visualizing mucus expression. (Kit 395B-Sigma-Aldrich). Images with 20 × magnification were used for the analysis. The area of the mucus and the total area of airway epithelial layer were measured using image J software and mucus percentage in epithelial layer was calculated using the following formula:

$${\text{Mucus }}\% \,\,{\text{in epithelial layer}}\, = \,{{{\text{sum of area of the mucus}}} \mathord{\left/
 {\vphantom {{{\text{sum of area of the mucus}}} {{\text{total area of the epithelial layer}}\,{\text{*}}\,100}}} \right.
 \kern-\nulldelimiterspace} {{\text{total area of the epithelial layer}}\,{\text{*}}\,100}}\,$$

Lung fibrosis from trichrome staining, small airway and arterial wall thickness were measured using image J software as previously described [24]. All the analysis was performed in a blinded manner.

### Biochemical assay

Lung protein extract was prepared using RIPA buffer that contained a mixture of proteinase and phosphatase inhibitors (Sigma, USA), protein concentration was measured using BCA kit (Sigma, USA). The content of Muc5ac in lung tissue lysate was measured using ELISA kit (Cloud-Clone-Crop, Houston, TX). The levels of Cysteinyl Leukotriene (Cayman Chemical, Ann Arbor, MI), IL-5 and IL-13 (both from R&D Sytems, Minneapolis, MN) in BAL were measured using ELISA kit followed by the manufacturer’s guide.

### Reverse-transcription PCR

Levels of mRNA expression for TNF-α and IL-1β were assessed in lung tissues by quantitative reverse-transcription polymerase chain reaction (q RT-PCR) as previously described with some modifications [24]. Briefly, total RNA was extracted from the lung tissues using a commercially available kit (RNAiso Plus, Takara, Japan). cDNA synthesis was performed with a High Capacity cDNA Reverse Transcription kit (Applied Biosystems, USA) on 2 mg RNA. For each RT-PCR reaction, 50 ng cDNA was used with TaqMan gene expression master mix kit (Applied Biosystems, USA). Primers were targeted against mice TNF-α (Applied Biosystems Gene Expression Assay, Mm00443258) and mice IL-1β (Applied Biosystems Gene Expression Assay, Mm00434228). All data were normalized against Eukaryotic 18 s rRNA endogenous control (FAM^™^/MGB pobe, non-primer limited) (Applied Biosystems Gene Expression Assay, ABI 4333760T) for each sample. Amplifications were performed in triplicate using an ABI Prism 7900HT Sequence Detection System (Applied Biosystems).

### Western blotting

Western blot experiments were performed as previously described [24]. Briefly, the lung protein extracts were separated using SDS-PAGE and transferred to PVDF membranes (BIO-RAD, USA). The blots were incubated with primary antibodies (1:1000) against: PDGF-BB (ab-23914) and α-SMA (ab-21027, abcam, Cambridge, Massachusetts), MMP-2 (sc-13594), MMP-9 (sc-21733), iNOS (sc-7271), PCNA (sc-25280), COX-2 (sc-7951), FGF-2 (sc-1360), ERK(sc-514302), p-ERK (sc-23759-R), Akt (sc-1619), p-Akt (sc-7985-R), VEGF (sc-53462), TGF-β1 (sc-146), and β-actin (sc-47778) (all from Santa Cruz Biotechnology, Santa Cruz, CA), followed by incubation with HRP-conjugated secondary antibody (1:2000, sc-2354, sc-2002, sc-2004, Santa Cruz, CA). Immunoreactivity was detected using an enhanced chemiluminescence Western blotting detection kit (Amersham, Piscataway, NJ). Results were quantified using Image J software.

### Statistical analysis

All data are reported as means ± SEM. Statistical differences were evaluated with a one-way ANOVA, followed by Bonferroni’s post hoc test using GraphPad Prism 5 (GraphPad Software, San Diego, CA). *P* ≤ 0.05 were considered to indicate statistically significant differences.

## Results

### BIBF1000 reduces allergen-induced lung inflammation and mediator expression

**Fig. 2:**
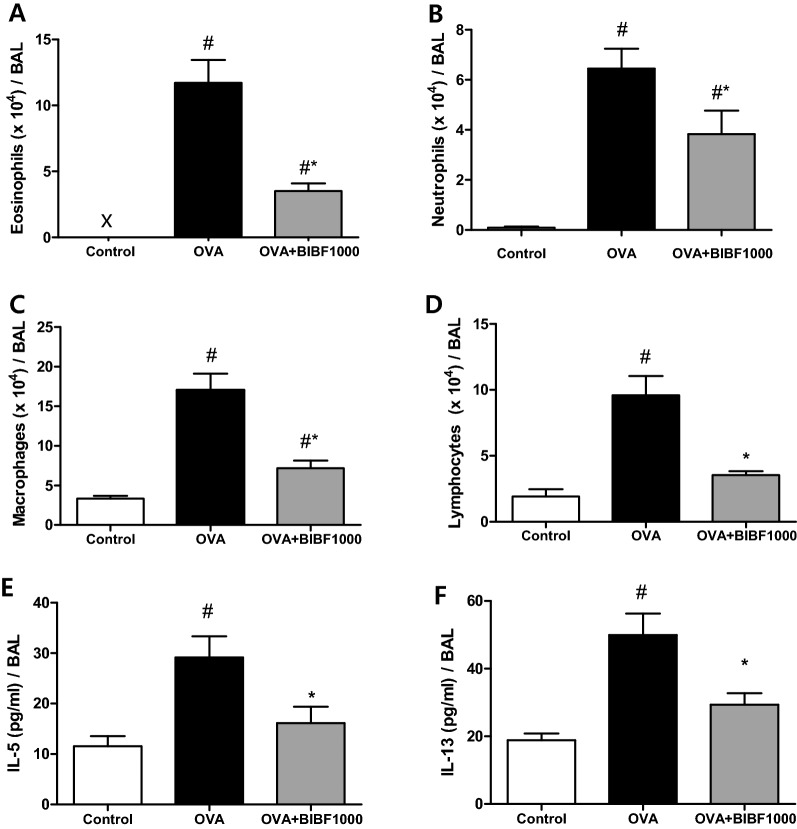
Inhibition of VEGFR, PDGFR, and FGFR signaling by BIBF1000 reduced lung inflammatory cell accumulation in a mouse model of ovalbumin (OVA) allergen-induced chronic asthma. Inflammatory cell counts for **A** eosinophils, **B** neutrophils, **C** macrophages, **D** lymphocytes, and levels of **E** IL-5 and **F** IL-13 in BAL fluids. All values are mean ± SEM, *n* = 8. #*p* < 0.05 vs. control, *p < 0.05 vs. vehicle

**Fig. 3:**
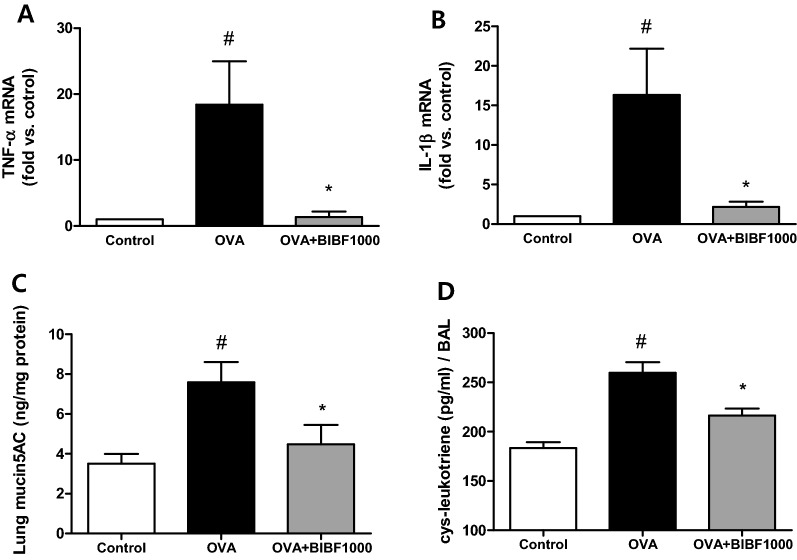
Treatment with BIBF1000 reduced: **A**, **B** allergen-induced lung tissue mRNA expression of TNF-α and IL-1β; **C** lung tissue MUC5AC content, and the contents of cys-leukotrienes in lavage fluid. All values are mean ± SEM, *n* = 8. #*p* < 0.05 vs. control, *p < 0.05 vs. vehicle

On day 120, there was a significant increase in inflammatory cell influx into the lungs of OVA-challenged mice compared to control mice (Fig. 2A–D). However, the numbers of eosinophils, macrophages, neutrophils, and lymphocytes in the BAL were reduced by 70.0%, 57.9%, 47.5%, and 63.0%, respectively, in mice treated with BIBF1000, compared to vehicle-treated mice (Fig. 2A–D). The levels of IL-5 and IL-13 in BAL were significantly lower in BIBF1000-treated mice, compared to vehicle controls (Fig. 2E, F). RT-PCR revealed a significant increase of proinflammatory cytokines TNF-α and IL-1β mRNA expression in OVA-challenged animal lungs. However, the mRNA expression of TNF-α and IL-1β was significantly reduced in mice treated with BIBF1000 (Fig. 3A, B). Furthermore, BIBF1000 treatment lowered lung mucin5AC content and BAL cys-leukotriene in mice compared to vehicle controls (Fig. 3C, D).

### BIBF1000 reduces airway remodeling and mucus expression

**Fig. 4:**
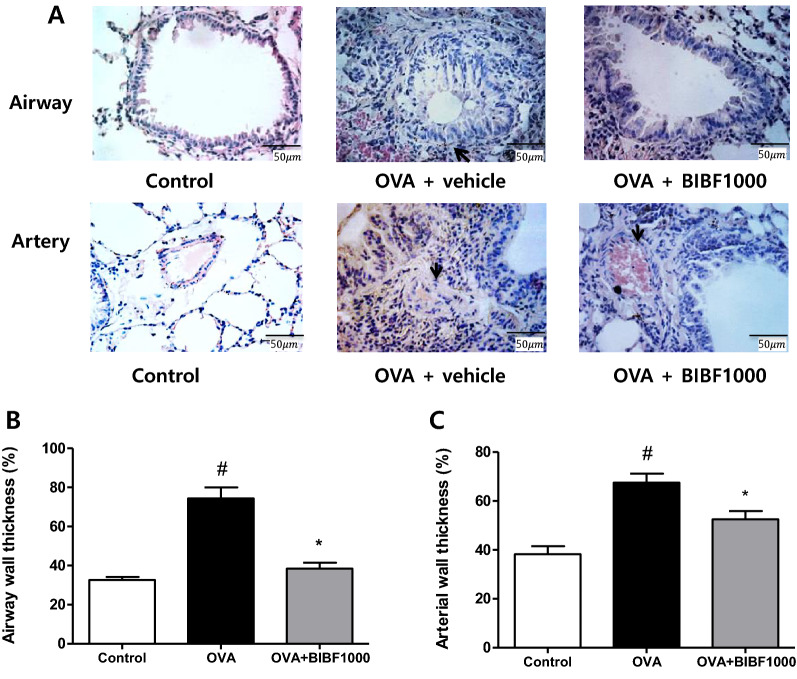
Treatment with BIBF1000 reduced allergen-induced airway and pulmonary arterial remodeling in mice. **A** Hematoxylin and eosin (HE) staining. **B** Airway wall thickness. **C** Pulmonary arterial wall thickness in each treatment group. All values are mean ± SEM, *n* = 8. #*p* < 0.05 vs. control, *p < 0.05 vs. vehicle

**Fig. 5:**
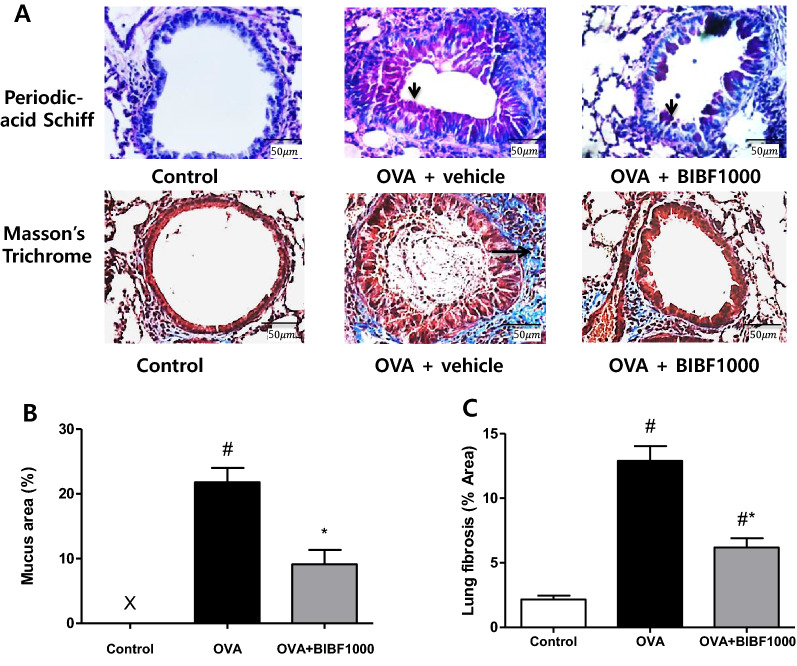
Treatment with BIBF1000 reduced the secretion of airway mucus and lung fibrosis. **A** Representative Periodic acid Schiff (PAS) staining for mucus and Masson’s trichrome staining for collagen deposition in airways. Quantitative analysis of: **B** mucus secretion and **C** lung fibrosis. All values are the mean ± SEM. *N* = 8. #*p* < 0.05 vs. the sham control group, *p < 0.05 vs. the vehicle group

Histological analysis revealed typical pathological features of chronic asthma. Repeated OVA challenge resulted in increased lung inflammatory cell infiltration, airway and pulmonary arterial wall thickening, collagen accumulation, and mucus expression (Figs. 4, 5). Treatment with BIBF1000 reduced airway and pulmonary arterial remodeling with a 48.3% and 22.2% reduction in small airway and small pulmonary arterial wall thickness, respectively, compared to vehicle-treated animals (Fig. 4). Histological analysis with PAS and Masson’s trichrome staining showed marked reduction in the amount of mucus secretion of goblet cells and collagen accumulation of peribronchial areas in mice treated with BIBF1000, compared to vehicle-treated mice (Fig. 5).

**Fig. 6:**
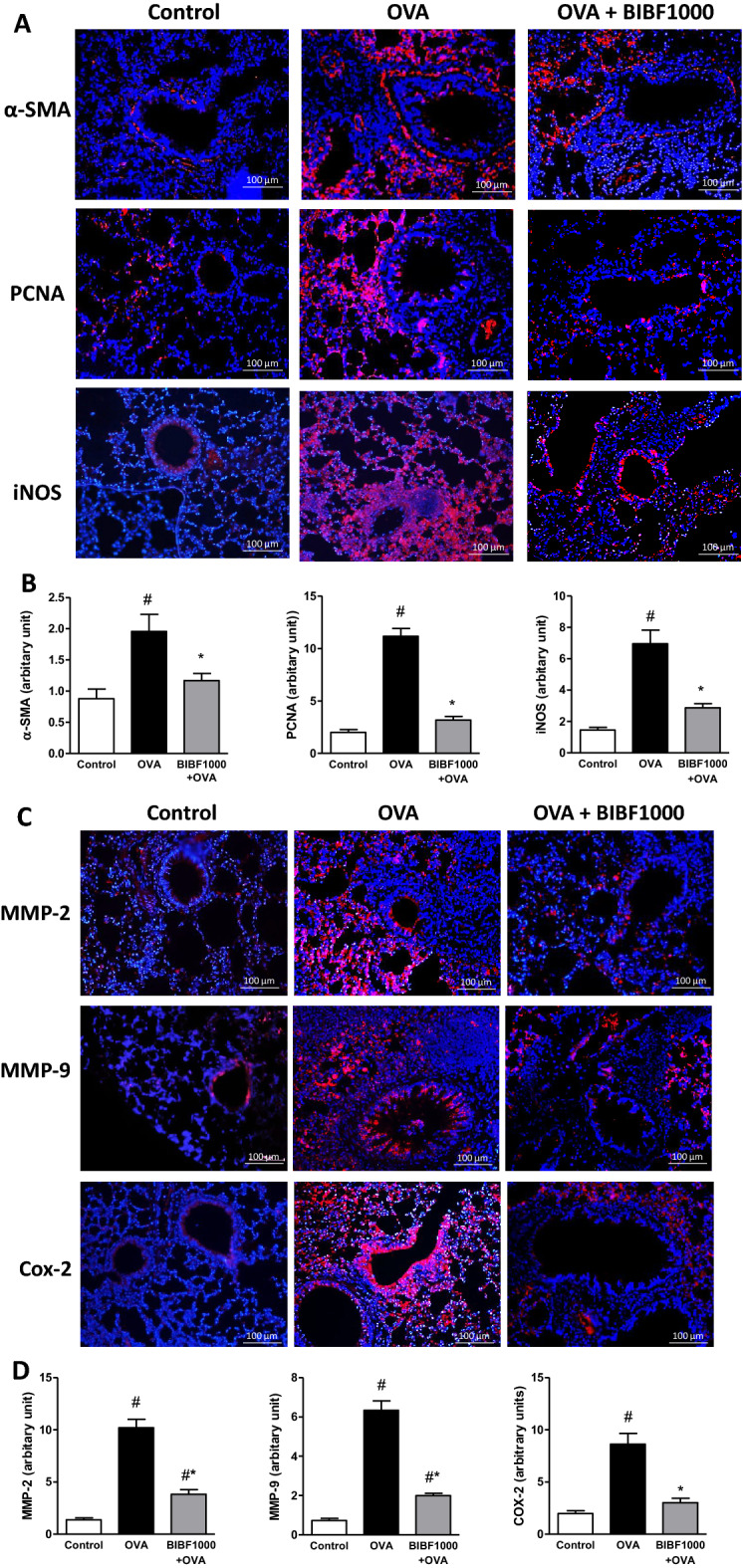
Lung tissue expressions of α-SMA, PCNA, iNOS, MMP-2, MMP-9, and COX-2 in allergen-induced chronic asthma in mice. Immunohistochemical analysis for the expression of **A**, **B** α-SMA, PCNA, and iNOS; **C**, **D** MMP-2, MMP-9, and COX-2 in lung tissues from each treatment group. All values are mean ± SEM, *n* = 8. #*p* < 0.05 vs. sham control, *p < 0.05 vs. vehicle

**Fig. 7:**
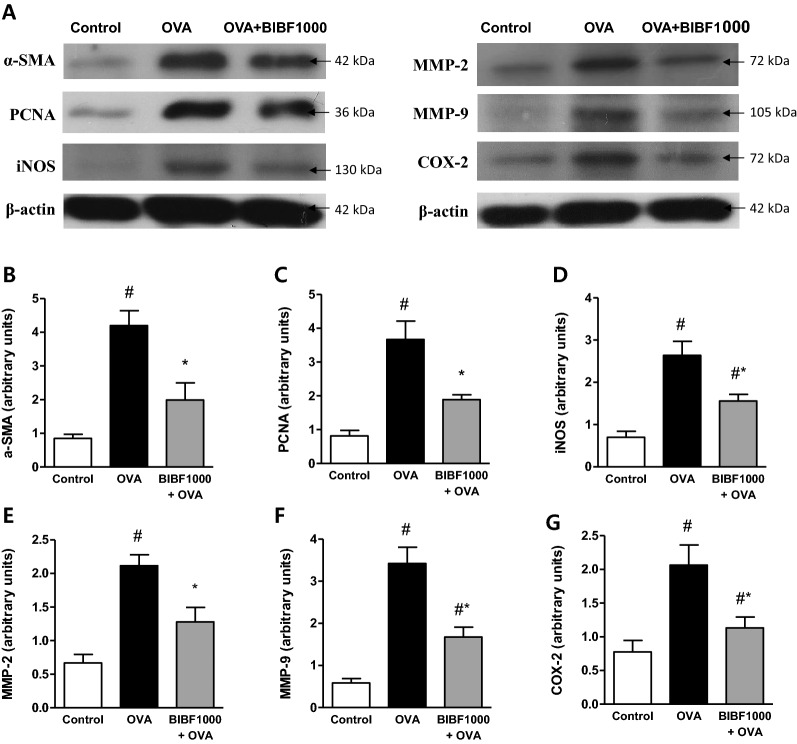
Treatment with BIBF1000 reduced allergen-induced expression of α-SMA, PCNA, iNOS, MMP-2, MMP-9, and COX-2 in an experimental model of chronic asthma in mice. **A** Representative western blots of the expression of α-SMA, PCNA, iNOS, MMP-2, MMP-9, COX-2, and β-actin in each study group. Mean densitometric analysis of immunoblots for **B** α-SMA, **C** PCNA, **D** iNOS, **E** MMP-2, **F** MMP-9, and **G** COX-2 in each study group. All values are expressed as mean ± SEM, *n* = 5. #*p* < 0.05 vs. sham control, *p < 0.05 vs. vehicle

Immunohistochemistry of vehicle-treated asthma mice demonstrated substantial cell proliferation in the thickened media layer of the airway. This layer was composed of α-smooth muscle actin- and PCNA-positive cells in vehicle-treated asthma lung tissues (Fig. 6A and B). Immunohistochemistry also showed that there was a marked increase in iNOS, MMP-2, MMP-9, and COX-2 in vehicle-treated asthma lungs (Fig. 6A–D). In contrast, treatment with BIBF1000 reduced smooth muscle cell proliferation and nitrosative stress (Fig. 6A, B). Similarly, the chronic asthma-induced expressions of MMP-2, MMP-9, and COX-2 were strongly suppressed by BIBF1000 treatment (Fig. 6C and D). Furthermore, western blot analysis further confirmed that BIBF1000 treatment attenuated the lung expression of α-SMA, PCNA, iNOS, MMP-2, MMP-9, and COX-2, compared to vehicle-treated asthma lung tissues (Fig. 7A–G).

### Effect of BIBF1000 on the expression of growth factors and signaling molecules

**Fig. 8:**
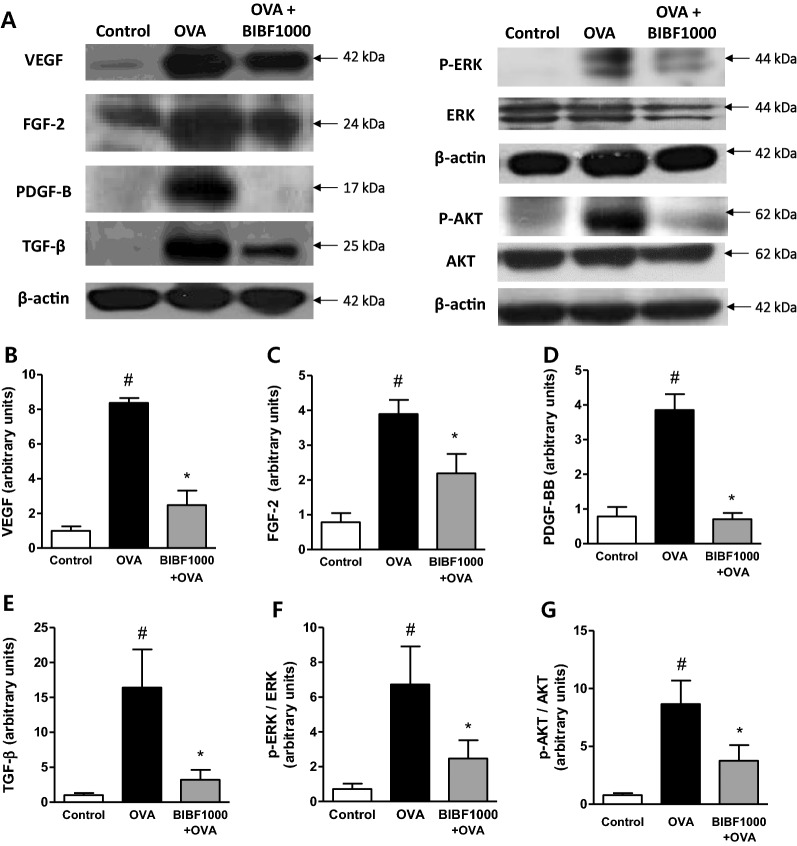
Treatment with BIBF1000 reduced allergen-induced expression of VEGF, FGF-2, PDGF, and TGF inhibited the phosphorylation of AKT and ERK in an experimental model of chronic asthma in mice. **A** Representative western blots of the expressions of VEGF, FGF-2, PDGF, TGF, p-ERK, ERK, p-AKT, AKT, and β-actin in each study group. Mean densitometric analysis of immunoblots for **B** VEGF, **C** FGF-2, **D** PDGF, and **E** TGF, **F** p-ERK/ERK, **G** p-AKT/AKT ratios in each study group. All values are expressed as mean ± SEM, *n* = 5, 6. #*p* < 0.05 vs. sham control, *p < 0.05 vs. vehicle

Western blot analysis showed increased levels of PDGF-BB, VEGF, FGF-2, and TGF-β in vehicle-treated asthma lungs, compared to the sham control group (Fig. 8A–E). The expressions of these growth factors were significantly reduced in mice treated with BIBF1000, compared to vehicle controls (Fig. 8A–E). Furthermore, BIBF1000 reduced the phosphorylation of ERK and AKT in OVA-challenged lungs (Fig. 8A, F and G).

## Discussion

The present study examined the anti-remodeling and anti- inflammatory effects of BIBF1000, a triple-tyrosine kinase inhibitor that targets VEGF, PDGF, and FGF receptor signaling in an experimental model of chronic asthma. We found that administration of BIBF1000 significantly reduced allergen-induced chronic inflammation, mucus expression, and airway remodeling. Furthermore, the protective effect afforded by BIBF1000 in reducing airway remodeling was associated with down-regulation of airway inflammation, and inflammatory mediators (MMP-2, MMP-9, COX-2, and iNOS), as well as inhibition of ERK and AKT phosphorylation.

The asthmatic airway is characterized by chronic inflammation, sub-epithelial fibrosis, airway mucus hypersecretion, and airway wall thickening [1–3]. Inflammatory cells, such as polymorphonuclear **(**PMN**)** leukocytes, are recruited to the airway after allergen exposure and during asthma exacerbations [1–3]. Many factors contribute to leukocytes recruitment to the airway, including cytokines and chemokines [1–5]. Moreover, the PMN leukocytes contribute to the development of airway inflammation and airway remodeling through release of granular enzymes, growth factors, and cytokines (i.e., TNF-α, IL-1β) [1–5]. VEGF receptor blockade ameliorated allergen OVA-induced eosinophilic inflammation and cytokine production [25]. Nintedanib inhibits macrophage activation and attenuates vascular remodeling in a mouse model of systemic sclerosis [26]. In the present study, treatment with BIBF1000 reduced allergen-induced inflammatory cell infiltration and cytokine expression (TNF-α, IL-1β) in the lung. Type 2 inflammation plays central roles in developing allergic asthma [27]. Each of the type-2 cytokines (interleukin (IL)-4, IL-5, and IL-13) exerts several roles in the airway inflammation cascade [27–30]. IL-4 plays a key role in Th2 cell differentiation [28], while IL-5 is specifically involved in eosinophilic inflammation and airway hyperresponsiveness [29]. However, IL-13 is known to be a powerful stimulator of eosinophilic inflammation, tissue fibrosis, mucus metaplasia, alveolar remodeling, and airways’ hyperresponsiveness [30]. In the present study, BIBF1000 reduced allergen OVA-induced IL-5 and IL-13 production in the lung. Furthermore, comparing to nintedanib, BIBF1000 was equally potent and effective in reducing OVA-induced airway inflammatory cell infiltration and cytokine production in mice [19]. These findings further support the hypothesis that VEGFR, PDGFR, and FGFR signaling may be important in mediating allergic airway inflammation.

Airway mucus can contribute to the airway obstruction seen in asthma. In humans, sub-epithelial glands are the major source of airway mucus [31]. Mucous cell metaplasia by which pleomorphic cells of the airway surface epithelium differentiate to become mucous cells is a critical event to the development of the mucus hypersecretion in chronic airway diseases [32]. Secretion of mucins from airway epithelial cells can be stimulated by a number of inflammatory mediators, including TNF-α, IL-13, leukotrienes, and prostaglandins [30, 33–36]. TNF-α has been shown to stimulate mucin release through activation of nitric oxide synthase, as well as via activation of NF-κB in a human lung epithelial cell [35, 36]. In the present study, treatment with BIBF1000 reduced OVA-stimulated production of TNF-α, IL-13, and leukotrienes, attenuated the expression of iNOS and COX-2 in mice. Furthermore, collagen degradation beneath the epithelium while permitting epithelial cells to migrate downward to form new or enlarge existing mucous glands is another important contributing factor to the development of the mucus hypersecretion in chronic airway diseases [32]. Matrix metalloproteinases’ (MMPs) activation contributes to the mucus hypersecretion via regulation of matrix deposition and degradation and thus promoting mucous metaplasia and gland enlargement in chronic airway diseases [32]. In the present study, treatment with BIBF1000 reduced mucus expression of goblet cells and lung MUC5AC content. The reduction of mucus expression in this study was attributed to inhibition of multi inflammatory mediators, including TNF-α, IL-1β, IL-13, MMP-2, MMP-9, iNOS, COX-2, and leukotriene production.

Airway wall thickening and sub-epithelial fibrosis have been associated with disease severity in chronic asthma. Compared to non-asthma controls, airway wall thickness is increased 50–300% in fatal asthma cases and 10–100% in cases of nonfatal asthma [37]. An increase in airway smooth muscle mass resulting from hypertrophy and hyperplasia is well described in the asthmatic airway [37]. Moreover, airway smooth muscle cells contribute to the perpetuation of tissue inflammation and are important sources of extracellular matrix (ECM) in the asthmatic airway [3]. Growth factors, including PDGF, VEGF, FGF, and TGF, activate receptors with intrinsic kinase activity and have been shown to promote airway smooth muscle cell proliferation, migration, and ECM accumulation [11–16]. Furthermore, transformation of fibroblasts to myofibroblasts has been implicated in the pathogenic events of airway remodeling [37, 38]. Myofibroblasts are major sources of cytokines, including TGF-β, which stimulates collagen production [38, 39]. BIBF1000 inhibited TGF-stimulated fibroblasts to myofibroblasts’ transformation and lung fibrosis [8]. In the present study, treatment with BIBF1000 reduced airway wall thickness and lung fibrosis. The reduction in airway wall thickness afforded by BIBF1000 treatment was accompanied by reduction in airway smooth muscle mass and cell proliferation, as well as reduction in the expression of PDGF-BB, VEGF, FGF-2, and TGF-β.

The mechanisms of airway remodeling are not well defined. A number of mediators have been implicated in the pathogenesis of airway remodeling [1–3]. Matrix metalloproteinases contribute to the pathogenesis of airway remodeling via their influence to alter the collagen, elastin, and other extracellular matrix proteins of the airways [23, 40]. Growth factors PDGF, FGF, and VEGF promote cell proliferation, migration, and tissue remodeling via stimulating MMPs activation, and phosphorylation of ERK and AKT signaling pathways [41–44]. In the present study, the expression of MMP-2 and MMP-9, and the phosphorylation of ERK and AKT induced by repeated OVA challenge were inhibited by BIBF1000 treatment.

Pulmonary arterial hypertension and chronic airway diseases (asthma, COPD) share important pathological features, including inflammation, smooth muscle proliferation, and remodeling [45]. In an experimental model of pulmonary arterial hypertension in rats, we showed that BIBF1000 prevented and reversed the progression of severe pulmonary arterial hypertension, inhibited pulmonary arterial neointimal formation, and improved survival [20]. Furthermore, this compound inhibited growth factor, and hypoxia-stimulated pulmonary arterial smooth muscle cell outgrowth from explant cultures of rat pulmonary arteries [20]. In the present study, small pulmonary arterial wall thickness was significantly increased following repeated OVA challenge. BIBF1000 treatment also significantly reduced pulmonary arterial wall thickness. These studies suggest that simultaneous inhibition of VEGF, PDGF, and FGF receptor signaling with BIBF1000 can target inflammatory cells, as well as lung structural cells, including inhibition of OVA-induced inflammatory cell infiltration, smooth muscle cells’ migration and proliferation, and transformation of fibroblasts to myofibroblasts, etc., thus ameliorating lung remodeling. Furthermore, BIBF1000 may have the potential to reverse the progression of pulmonary remodeling. Further studies are warranted.

## Conclusions

Administration of BIBF1000, an orally active small molecule triple-tyrosine kinase inhibitor that targets VEGF, PDGF, and FGF receptor signaling, significantly reduced allergen-induced chronic airway inflammation, mucus expression, airway, and pulmonary arterial remodeling. The protective effect afforded by BIBF1000 in reducing airway and arterial remodeling was associated with down-regulation of growth factors, inflammatory mediators (Il-5, Il-13, TNF-α, IL-1β, MMP-2, MMP-9, COX-2, and iNOS), as well as inhibition of ERK and AKT phosphorylation.

## Data Availability

The datasets used and/or analyzed during the current study are available from the corresponding author on reasonable request.

## Abbreviations

OVA: Ovalbumin
BAL: Bronchoalveolar lavage
COPD: Chronic obstructive pulmonary disease
PAH: Pulmonary arterial hypertension
FGF: Basic fibroblast growth factor
PDGF: Platelet-derived growth factor
VEGF: Vascular endothelial growth factor
TGF-β: Transforming growth factor-β
RTK: Tyrosine kinase receptor
BSMC: Bronchial smooth muscle cell
IPF: Idiopathic pulmonary fibrosis
PAS: Periodic acid Schiff
MMP: Matrix metalloproteinase
MUC5AC: Mucin 5AC
iNOS: Inducible nitric oxide synthase
PMN: Polymorphonuclear
ECM: Extracellular matrix
COX-2: Cyclooxygenase 2
